# Actual Use of Multiple Health Monitors Among Older Adults With Diabetes: Pilot Study

**DOI:** 10.2196/15995

**Published:** 2020-03-23

**Authors:** Yaguang Zheng, Katie Weinger, Jordan Greenberg, Lora E Burke, Susan M Sereika, Nicole Patience, Matt C Gregas, Zhuoxin Li, Chenfang Qi, Joy Yamasaki, Medha N. Munshi

**Affiliations:** 1 School of Nursing University of Pittsburgh Pittsburgh, PA United States; 2 Joslin Diabetes Center Boston, MA United States; 3 Research Services Boston College Chestnut Hill, MA United States; 4 Carroll School of Management Boston College Chestnut Hill, MA United States; 5 North Shore Medical Center Salem Hospital Salem, MA United States; 6 Hollywood Presbyterian Medical Center Los Angeles, CA United States; 7 Beth Israel Deaconess Medical Center Boston, MA United States; 8 Harvard Medical School Boston, MA United States

**Keywords:** mobile health, aged, lifestyle, self-management, diabetes mellitus, type 2

## Abstract

**Background:**

Previous studies have reported older adults’ perceptions of using health monitors; however, no studies have examined the actual use of multiple health monitors for lifestyle changes over time among older adults with type 2 diabetes (T2D).

**Objective:**

The primary aim of this study was to examine the actual use of multiple health monitors for lifestyle changes over 3 months among older adults with T2D. The secondary aim was to explore changes in caloric intake and physical activity (PA) over 3 months.

**Methods:**

This was a single-group study lasting 3 months. The study sample included participants who were aged ≥65 years with a diagnosis of T2D. Participants were recruited through fliers posted at the Joslin Diabetes Center in Boston. Participants attended five 60-min, biweekly group sessions, which focused on self-monitoring, goal setting, self-regulation to achieve healthy eating and PA habits, and the development of problem-solving skills. Participants were provided with the Lose It! app to record daily food intake and devices such as a Fitbit Alta for monitoring PA, a Bluetooth-enabled blood glucose meter, and a Bluetooth-enabled digital scale. Descriptive statistics were used for analysis.

**Results:**

Of the enrolled participants (N=9), the sample was white (8/9, 89%) and female (4/9, 44%), with a mean age of 76.4 years (SD 6.0; range 69-89 years), 15.7 years (SD 2.0) of education, 33.3 kg/m^2^ (SD 3.1) BMI, and 7.4% (SD 0.8) hemoglobin A_1c_. Over the 84 days of self-monitoring, the mean percentage of days using the Lose It!, Fitbit Alta, blood glucose meter, and scale were 82.7 (SD 17.6), 85.2 (SD 19.7), 65.3 (SD 30.1), and 53.0 (SD 34.5), respectively. From baseline to completion of the study, the mean daily calorie intake was 1459 (SD 661) at week 1, 1245 (SD 554) at week 11, and 1333 (SD 546) at week 12, whereas the mean daily step counts were 5618 (SD 3654) at week 1, 5792 (SD 3814) at week 11, and 4552 (SD 3616) at week 12. The mean percentage of weight loss from baseline was 4.92% (SD 0.25). The dose of oral hypoglycemic agents or insulin was reduced in 55.6% (5/9) of the participants.

**Conclusions:**

The results from the pilot study are encouraging and suggest the need for a larger study to confirm the outcomes. In addition, a study design that includes a control group with educational sessions but without the integration of technology would offer additional insight to understand the value of mobile health in behavior changes and the health outcomes observed during this pilot study.

## Introduction

### Background

A total of 25.2% or 12 million older Americans (aged ≥65 years) have type 2 diabetes (T2D) [1,2]. Older adults are at substantial risk for acute and chronic microvascular and cardiovascular complications related to T2D, which is linked to higher mortality [2]. Older adults also endure many daily burdens associated with T2D management, for example, complex lifestyle management [3], adherence to medication [4,5], psychological effects [6,7], and financial impact [8,9]. In 2017, the direct and indirect costs attributed to T2D in the United States were US $327 billion [10].

Lifestyle intervention, which focuses on decreasing energy intake and increasing physical activity (PA), is the most efficient nonmedical approach to self-management of T2D [11]. At present, achieving goals for dietary intake [12,13] and PA [1,14,15] remains challenging for many older adults with T2D. In a study with a large and diverse cohort of older adults with T2D (n=2400) from 16 US clinical sites, only a small percentage of older participants implementing intensive lifestyle interventions (33.7% at 1 year and 21.4% at 4 years) achieved or exceeded the national PA threshold for improved health (ie, ≥150 min per week), and a smaller percentage of older participants (20.2% at 1 year and 11.3% at 4 years) met the PA threshold of the American College of Sports Medicine (ie, ≥250 min per week) [15]. Moreover, older adults with T2D face challenges achieving dietary intake goals [12,13]. One study showed that more than 50% of older adults with T2D did not consider diet as part of diabetes management [13].

Numerous available and emerging technologies, such as wearable trackers, smartphone apps, and remote monitoring devices, can help older adults make lifestyle changes. One study demonstrated that wearable trackers and telehealth platforms can encourage older adults with hypertension to engage in healthier lifestyles [16]. Furthermore, a literature review published in 2018 [17] indicated that more than 60% of elderly people were interested in the future use of wearable devices and the devices’ potential to improve PA. Similar findings were found from two cross-sectional surveys [18,19] and a qualitative study [20]; these noted that older adults were willing to use health monitors to track health information. However, the limitation of the reported studies is that they only assessed older adults’ perceptions of using health monitors; they did not examine older adults’ actual use of multiple health monitors for complex lifestyle changes over time.

### Objective

The primary aim of this study was to examine the actual use of multiple health monitors for lifestyle changes over 3 months among older adults with diabetes. The secondary aim was to explore changes in caloric intake and PA over 3 months.

## Methods

### Study Design

**Figure 1:**
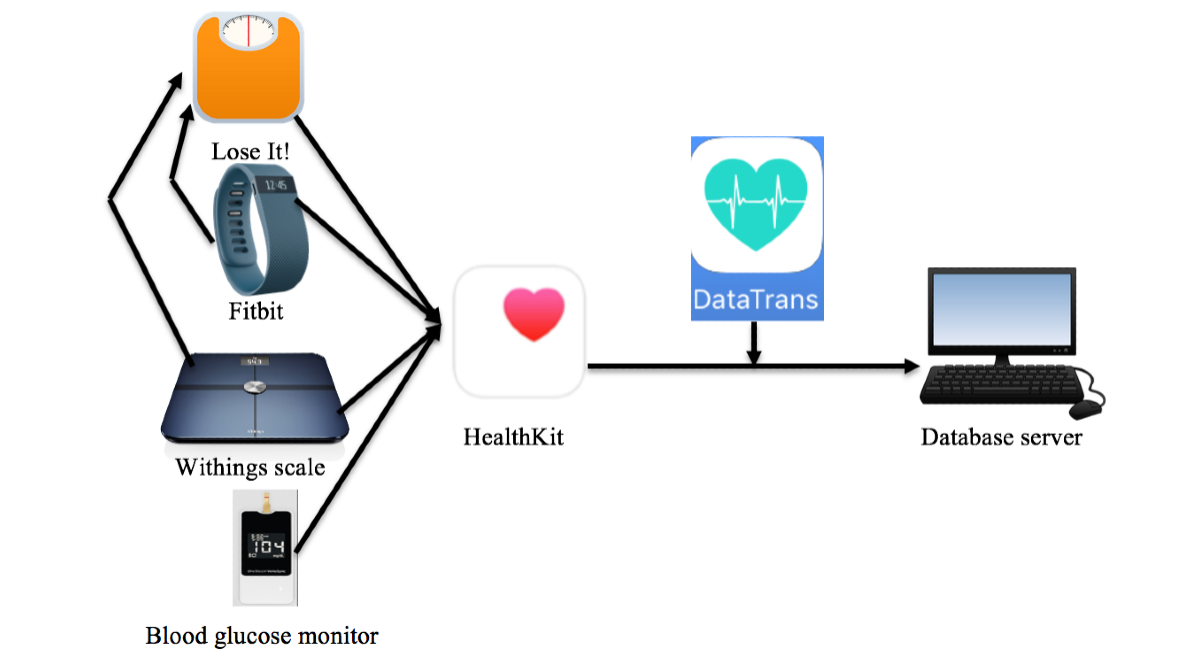
The infrastructure supporting remotely transmitted data collection.

This was a single-group study lasting 3 months. The study was approved by the institutional review board at Boston College and the Joslin Diabetes Center in Boston. All participants provided informed consent and were given monitoring app and devices, including Lose It!, a self-monitoring smartphone app to record daily food intake; a Fitbit Alta for monitoring PA; a Bluetooth-enabled blood glucose meter; and a Bluetooth-enabled digital self-weighing scale. Participants also received a OneTouch Verio Flex meter and OneTouch Verio test strips for blood glucose monitoring. Data from all the provided monitoring devices were transmitted to the research center by synchronizing the device and app data from the HealthKit and then synchronizing to the DataTrans app (Figure 1). In addition, participants attended five 60-min, biweekly group sessions, which focused on self-monitoring, goal setting, self-regulation of behavior to support healthy eating and PA habits, and the development of problem-solving skills.

#### Recruitment, Participants, and Settings

All participants were recruited through posted fliers from the Joslin Diabetes Center in Boston. Eligibility criteria included (1) previously diagnosed with T2D for 2 years or more, (2) aged 65 years or older, (3) BMI between 27 and 40 kg/m^2^, (4) availability of wireless internet service at home, (5) prescribed insulin or oral medications for 1 year or more, (6) no changes in medications for 6 months or more before enrolling in the study, (7) used the Lose It! app on their computer or smartphone for the 5-day practice period, and (8) able to read and speak in English.

Individuals were excluded if they (1) were planning to frequently travel, vacation, or relocate within the next 6 months; (2) were unable to walk two blocks or had a lower limb amputation, severe arthritis, or other medical condition that prevented walking for exercise; (3) had severe complications of diabetes that interfered with self-management skills, such as renal disease (albumin/creatinine >300 µg/mg), severe peripheral diabetic neuropathy, severe peripheral vascular disease, symptomatic autonomic neuropathy, recent myocardial infarction (within the last 6 weeks), congestive heart failure, or other severe cardiac disease, or severe hypertension (systolic blood pressure >160/90 mm Hg); (4) were receiving current treatment for a serious mental illness (eg, schizophrenia, bipolar disorder, substance abuse, or eating disorders); (5) had severe visual, hearing, or cognitive impairments (eg, dementia and intellectual disability); or (6) were unable or unwilling to use the technology toolkit for data collection. All participants were required to obtain clearance from their primary care provider (PCP) before enrollment.

#### Protocol of Group Sessions

The five group sessions focused on (1) self-monitoring and goal setting, (2) healthy eating, (3) PA, (4) self-regulation of behavior to support healthy eating and PA habits, and (5) the development of problem-solving skills. All participants were taught to use self-monitoring devices 1 to 2 weeks before starting the group sessions. During the first group session, individualized goals for caloric intake were identified. In addition, participants were trained to observe how their weight changes corresponded with changes in dietary intake and PA. Participants were also counseled on monitoring their blood glucose at different times to identify when their blood glucose would be out of range, and they were taught to implement walking and dietary changes at particular times to improve blood glucose levels that were out of range. Such self-regulation skills were reinforced with guidance that aided in the development of the participants’ problem-solving skills. Suggestions for changes in medication regimens, particularly insulin or hypoglycemic agents, were not part of the group sessions but were communicated to the participants’ diabetes care providers. No intervention related to medication was performed by the study staff.

### Measurements

#### Sociodemographic Data and Medical History

These data were collected using the self-administered sociodemographic and lifestyle questionnaire, which consists of 25 primary questions designed to assess standard sociodemographic and socioeconomic information, such as age, gender, marital status, education, employment status, income, and ethnicity or racial background. BMI (kg/m^2^) was calculated according to baseline weight and height. Baseline weight was measured using a digital scale (Tanita Corporation of America, Inc). Participants were asked to wear lightweight clothing while standing barefoot on the scale’s footpads. Height (cm) was measured with a stadiometer. Information on self-reported medical conditions, including hypertension, hyperlipidemia, diabetes, coronary artery disease, myocardial infarction, congenital heart disease, stroke, and congestive heart failure, was also collected. Cognitive status was assessed using the Montreal Cognitive Assessment, a brief screening tool for detecting cognitive dysfunction [21]. A total score of less than 26 typically indicates mild or more severe cognitive impairment (eg, dementia), whereas a total score of greater than or equal to 26 indicates intact cognition [22]. The information regarding medication changes was derived from the electronic medical records.

#### Actual Use of Technology

Participants’ use of the provided Lose It! app, Fitbit Alta, self-weighing scale, and glucose meter was objectively determined based on date-stamped information; each day of use was coded binarily (use vs nonuse). The number of days per week that each device was used was then calculated. In addition, the proportion of participants who used each self-monitoring app and device per day was calculated.

#### Weight, Steps, and Energy Intake

Objectively assessed daily body weight data were transmitted via the provided Bluetooth-enabled scales. Daily weights were then used to calculate the percentage weight change relative to the baseline weight. Objectively assessed daily steps and self-reported daily energy intake were transmitted to the research server via the Fitbit Alta and Lose It! app.

### Statistical Analysis

Descriptive statistics for continuous variables, such as age, BMI, and percentage change in weight, were reported as mean (SD). Categorical variables, such as gender, race, education, employment, and household income, were described using frequency counts and percentages. Descriptive analyses were conducted using SPSS Statistics version 25 (IBM Corp).

## Results

Among 14 approached participants, 5 were not eligible and 9 completed the study. The reasons for ineligibility (2/14, 14%) included difficulty commuting or parking and difficulty learning how to use the Lose It! app (2/9, 14%), fear of falling because of a health condition that prevented exercise (1/9, 7%), and personal circumstances (eg, sick family member and time conflict). All 9 participants attended all five group sessions.

Table 1 provides a description of the study sample (N=9). Most participants (8/9, 89%) were white, and 44% (4/9) participants were female. Participants were aged, on average, 76.4 years (SD 6.0; range 69-89 years), with 15.7 (SD 2.0) years of education. Participants were obese, with a mean BMI of 33.3 kg/m^2^ (SD 3.1) and hemoglobin A_1c_ of 7.4% (SD 0.8; range 5.8%-8.6%). The mean duration of T2D was 14.4 years (SD 8.1). In addition to T2D, many participants had high blood pressure (8/9, 89%) and hyperlipidemia (6/9, 67%).

**Figure 2:**
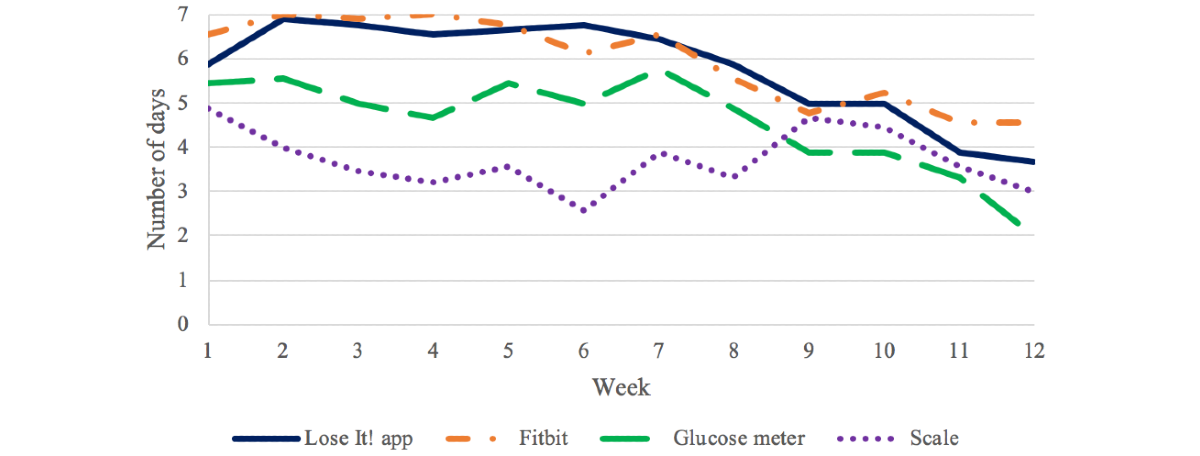
Number of days of using self-monitoring devices and app by week.

Over the 12 weeks (84 days) of self-monitoring, the mean percent days of using the Lose It!, Fitbit Alta, blood glucose meter, and scale were 82.7 (SD 17.6), 85.2 (SD 19.7), 65.3 (SD 30.1), and 53.0 (SD 34.5), respectively. Figure 2 illustrates the mean number of days of self-monitoring by week. Over 12 weeks, participants consistently used the Lose It! app daily from the beginning of the study to week 6, but then there was a gradual decrease to 4 days per week at the end of the study. A similar pattern was seen in the Fitbit Alta use. There was a decline in the use of the glucose meter over 12 weeks, starting from 5.7 days per week to 2 days per week. Scale use started at 5 days per week, decreased to 2.6 days per week by week 6, increased to 4.7 by week 9, and then decreased to 3 days by week 12.

**Table 1:** Sample description (N=9).

Demographic characteristics		Values
Age (years), mean (SD)		76.4 (6.0)
Education (years), mean (SD)		15.7 (2.0)
Duration of type 2 diabetes (years), mean (SD)		14.4 (8.1)
BMI (kg/m^2^), mean (SD)		33.3 (3.1)
Hemoglobin A_1c_ (%), mean (SD)		7.4 (0.8)
**Gender, n (%)**		
	Male	5 (56)
	Female	4 (44)
**Race, n (%)**		
	White (non-Hispanic)	8 (89)
	African American	1 (11)
**Employment, n (%)**		
	Employed	2 (22)
	Unemployed	7 (78)
**Marital status, n (%)**		
	Married or living with other	6 (67)
	Widowed, divorced, or other	3 (33)
**Income, n (%)**		
	<US $50,000	4 (44)
	≥US $50,000	5 (56)
**Medical conditions, n (%)**		
	Hypertension	8 (89)
	Hyperlipidemia	6 (67)
	Heart problems	3 (33)
	Fatty liver	3 (33)
	Gout	3 (33)
	Osteoarthritis	2 (22)
	Mild cognitive impairment	4 (44)

**Figure 3:**
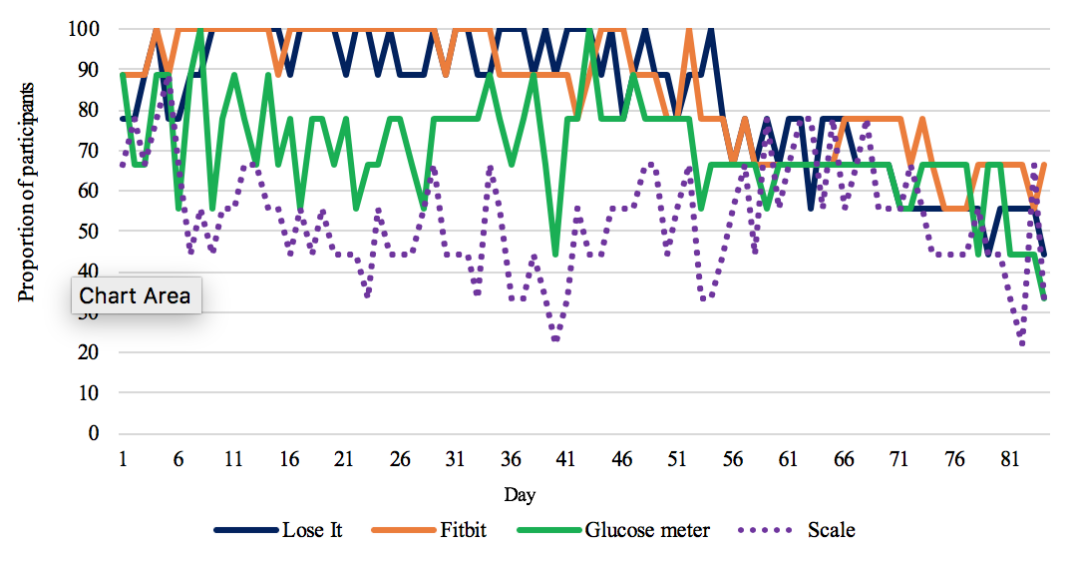
Proportion of participants using the self-monitoring device and app by day of monitoring.

Figure 3 illustrates the proportion of participants using each of the self-monitoring devices each day. All participants used the Lose It! app for tracking their food intake and Fitbit Alta for tracking their PA during the first 8 weeks (56 days), and 60% to 80% of the sample continued using the 2 devices after the first 8 weeks. The proportion of the participants who used the glucose meter and the scale was lower than that of the participants who used the Lose It! app and the Fitbit Alta during the first 8 weeks; however, the proportion of the participants who used the glucose meter and the scale was similar to that of the participants who used the Lose It! app and Fitbit Alta after 8 weeks.

**Figure 4:**
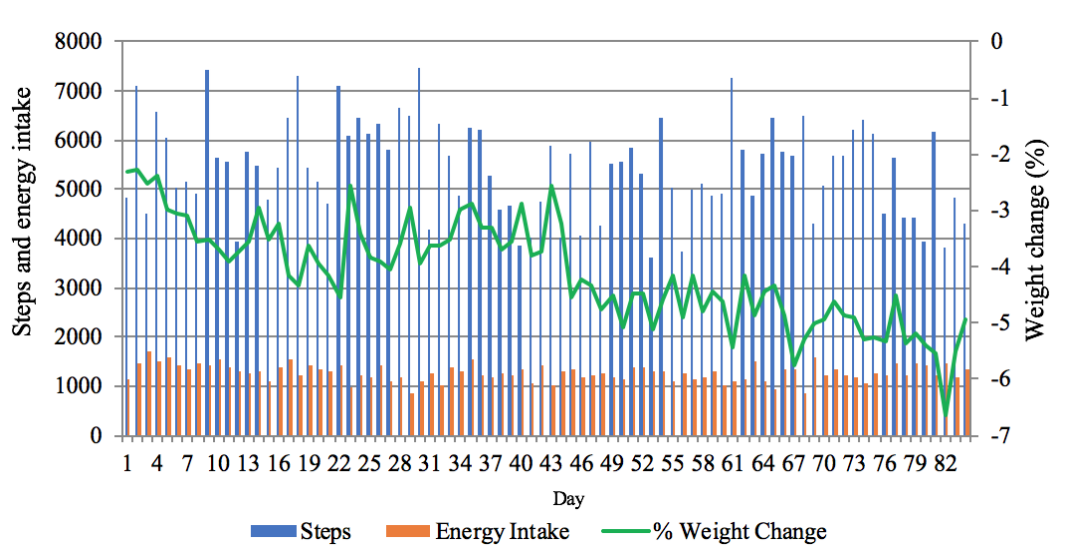
Energy intake, steps, and percent weight loss relative to baseline by day of monitoring.

From baseline to completion of the study (Figure 4), the mean caloric intake was 1459 (SD 661) at week 1, 1245 (SD 554) at week 11, and 1333 (SD 546) at week 12, whereas the daily step counts were 5618 (SD 3654) at week 1, 5792 (SD 3814) at week 11, and 4552 (SD 3616) at week 12. The mean percent weight loss from baseline to 12 weeks was 4.92% (SD 0.25). The dose of oral hypoglycemic agents or insulin was reduced in 5 participants by their diabetes care providers, 4 participants’ insulin prescriptions were reduced by 6 to 24 units and 1 participant’s oral hyperglycemic agent prescription was reduced to a smaller dose.

## Discussion

### Principal Findings

We conducted a single-group study to determine the usability of multiple health monitors among older adults with T2D. Our study participants were able to use multiple mobile health (mHealth) technology that is commercially available over a 12-week period to manage their daily lifestyle behaviors related to diet and PA. By leveraging multiple mHealth monitors, combined with group sessions, the results from our pilot study showed that improved lifestyle habits resulted in a healthy weight loss and reduced doses of insulin or other medications. Although this pilot study had a small sample size, the results are encouraging and suggest the need for a larger study to confirm the outcomes.

### Comparison With Prior Work

The findings from this study are consistent with the reported research [23-25], which has demonstrated that older adults with T2D are willing to learn and use technology for disease management. Moreover, this study provides unique evidence that the older adults can use multiple devices. In particular, all participants in our sample used the Lose It! app for diet tracking and Fitbit Alta for PA tracking every day, particularly during the first 8 weeks of the study. One possible explanation for adherence is that using these 2 monitoring devices may have helped participants understand what their current diet and PA levels were compared with the goals they set with their health care team and how changes made to their diet and PA directly impacted their weight and blood glucose levels. Another possible explanation is that attendance to the biweekly group sessions, which included discussions about self-monitoring, healthy eating, PA, self-regulation skills, and problem solving, was beneficial in motivating participants to use the provided tools to support their lifestyle changes. The mean number of days participants used the scale was lower compared with the usage of the other devices. The suspected reason for missing weight data is loss of wireless internet connection; several participants reported that weight data did not transfer to their smartphone, although they did step on the scale.

Our study found that the use of mHealth by older adults with T2D resulted in lifestyle changes. This might be because of two potential reasons. First, our study guided participants’ lifestyle behavior changes by using mHealth monitors to self-regulate and develop problem-solving skills [26,27]. Second, certain features of health monitors could offset some of the challenges of caring for an aging population that may face difficulty achieving goals for multiple lifestyle changes. For example, participants were able to compare current dietary intake with goals by simply reviewing the bar or pie graphs that are automatically generated in the Lose It! app. The graphical displays may have enabled participants to more clearly see progress regarding adherence to their dietary goals [28] and may have been particularly useful for those with health and numeric literacy concerns [29].

### Limitations and Strengths

The study has three main limitations; therefore, the results of this pilot study should be carefully considered. Owing to the small sample size, participants may not represent an older adult population with T2D cared for by community providers. The study should be replicated in an older population with T2D cared for by PCPs. Moreover, 2 participants in our study showed difficulty learning how to use the Lose It! app during the recruitment stage. As using a commercially available diet app such as Lose It! for diet monitoring might be relatively complex for some older populations, this issue needs to be further explored. Second, this was a single-group study, which lacked control of confounders for the behavior or outcome changes; however, the study’s aim was to examine the usability of multiple health monitors for lifestyle changes. A future study using a randomized controlled design is needed to explore if and how the use of mHealth devices is more efficient in achieving improved T2D self-management outcomes compared with the standard self-management approach. Third, a majority of the participants were white and had a relatively high education level. The results might not be generalizable to other populations. However, our study has a unique strength: it is the first to demonstrate the daily use of multiple mHealth devices for diabetes management in older adults.

### Conclusions and Implications

Although this pilot study had a small sample size, the results are encouraging and suggest the need for a larger study with a control group to confirm the outcomes. Future research should examine the motivation of older adults to use mobile apps to better self-manage their diabetes. A qualitative study of participants would collect valuable information to understand the role of social support in the context of the group setting in adopting and adhering to the use of new technologies. A study design that includes a control group with educational sessions without the integration of technology would offer additional insight to understand the value of mHealth in behavior changes and health outcomes observed during this pilot study.

## Abbreviations

mHealth: mobile health
PA: physical activity
PCP: primary care provider
T2D: type 2 diabetes
